# Effects of Aquatic Exercise in Older People with Osteoarthritis: Systematic Review of Randomized Controlled Trials

**DOI:** 10.3390/geriatrics10010012

**Published:** 2025-01-13

**Authors:** Carlos Ayán-Pérez, Daniel González-Devesa, Beatriz Montero-García, Silvia Varela

**Affiliations:** 1 Well-Move Research Group, Galicia Sur Health Research Institute (IIS Galicia Sur), Servicio Galego de Saúde-Universidade de Vigo, 36310 Vigo, Spain; cayan@uvigo.gal (C.A.-P.); silviavm@uvigo.gal (S.V.); 2 Department of Special Didactics, Universidade de Vigo, 36005 Pontevedra, Spain; b.monterogarcia46@gmail.com; 3 Grupo de Investigación en Actividad Física, Educación, y Salud (GIAFES), Universidad Católica de Ávila, C/Canteros, 05005 Ávila, Spain

**Keywords:** osteoarthritis, aquatic exercise, older people, water-based exercise, physical function

## Abstract

**Objective:** In this study, the objective is to analyze the efficacy of different aquatic physical exercise programs in the treatment of osteoarthritis in older people. **Material and Methods:** The systematic review was conducted until April 2024 and updated in November 2024 in five electronic databases. Randomized controlled studies in people over 60 years of age with a diagnosis of osteoarthritis were included. Water-based exercise interventions were assessed for their impact on osteoarthritis symptoms, walking ability, muscle strength, depressive symptoms, range of motion, body composition, fear of falling, fall risk, quality of life, range of motion, and dual task function. The methodological quality of included studies was assessed using the Physiotherapy Evidence Database. **Results:** A total of 12 studies were included, with sample sizes ranging from 35 to 312 participants. The presence of osteoarthritis in the sample was manifested in different joints. The methodological quality of the included studies varied from fair to good. Based on the results of this review, aquatic exercise significantly improved balance, stiffness, pain, and walking ability compared to non-exercise controls (*p* < 0.05). Compared with land-based exercise groups, only one study found significant differences between the two groups. **Conclusions:** Water-based exercise programs do not provide significantly superior benefits compared to other exercise modalities and appear to have limited effectiveness in the management of OA. However, the feasibility and high adherence make these programs a recommendable option for older people with OA. Future studies should investigate the impact of exercise intensity on the short- and long-term efficacy of aquatic therapy.

## 1. Introduction

Osteoarthritis (OA) is a chronic and progressive musculoskeletal disease and a major cause of pain and functional limitation [1]. It is characterized by complex disorders of the synovial joint, such as structural defects of the hyaline articular cartilage, loss of subchondral bone, and synovial hyperplasia or instability of tendons and ligaments [2]. The main symptoms of OA include joint pain, stiffness and limitation of movement. The progression of the disease is gradual and slow but can lead to joint failure and disability [3].

In 2020, approximately 595 million people worldwide had OA [4], and the incidence of OA is expected to increase significantly in the coming decades due to an ageing population [5]. Age is considered to be a major risk factor for the development of OA, and it is estimated that 9.6% of men and 18% of women over the age of 60 suffer from OA worldwide [6].

Currently, there is no known cure for OA. Available treatment options include intra-articular injections, surgical procedures, or pharmacological therapies although their efficacy has not been shown to be superior to placebo [7]. However, non-pharmacological and non-surgical approaches, particularly exercise, show an advantage over these in that they target physical function [5]. OA is the leading cause of disability in older people, leading to loss of functional ability, autonomy, and reduced quality of life [6]. Regular exercise can help improve movement ability as well as long-term pain reduction in adults with arthrosis [8]. In addition, it has been shown to contribute to improved physical fitness in older people [1]. These benefits translate into improved quality of life by facilitating the activities of daily living [9] and by reducing pain levels through the promotion of an active lifestyle [10]. Among the various training modalities, aquatic exercise is presented as a very interesting alternative, which has been shown to be effective in adults with chronic musculoskeletal conditions [11]. The buoyancy provided by water reduces the pressure of gravity on the muscles and joints [12]. As a result of submersion up to the umbilical area, approximately 50% of the body weight is unloaded, which reduces muscle fatigue in the areas most affected by OA, such as the hips and knees [13], making it easier to perform large and efficient movements [14]. In addition, aquatic exercise relieves pain and improves balance, strength, cardiorespiratory fitness, and quality of life [11]. Furthermore, it has been demonstrated to have a beneficial impact on mood and anxiety, while simultaneously reducing the likelihood of falls, thereby establishing a secure environment for exercise in older individuals with osteoarthritis [15]. It is important to keep in mind that aquatic exercise may have certain limitations, such as accessibility or financial costs, which could make it difficult for older people with OA to participate.

A number of reviews have been published on the effects of aquatic exercise in people with osteoarthritis [16]. However, none have focused specifically on older people so their findings may be inconsistent with respect to specific outcomes such as symptomatology, strength, or quality of life. A Cochrane review [17] detailed that the average age of the sample was 68 years. However, a closer look at the studies included in that review shows that in several of them, the age range of the sample was quite wide (40–89 years) including middle-aged adults. When compared with those in the early stages or with a relatively early onset, osteoarthritis affects older people more severely and has a greater impact [18,19]. Therefore, physical exercise prescriptions should be tailored to the characteristics of this population, taking into account their specific needs.

Several of the unique characteristics of exercise in an aquatic environment (reduced joint impact, reduced risk of falls, ease of movement, etc.) have been shown to be beneficial in the treatment of OA. Research conducted in the adult population suffering from OA has found positive results. There has, however, been no systematic review dedicated exclusively to older people. The aim in this study is to investigate the efficacy of different aquatic physical exercise programmes in the treatment of OA in older people. We also hope to provide a reference for future practical applications.

## 2. Materials and Methods

The method of reporting for this systematic review was based on the Preferred Reporting Items for Systematic Reviews and Meta-Analyses (PRISMA) guidelines [20]. This review was registered with the Open Science Framework (OSF), https://doi.org/10.17605/OSF.IO/9RQ86 (accessed on 3 September 2024).

### 2.1. Search Strategy

Five electronic databases (PubMed, Web of Science, Scopus, SportDiscus, and the Cochrane Central Register of Controlled Trials) were reviewed from their inception to April 2024 and updated in November 2024. Key search words and MeSH terms used in this review included: “osteoarthritis” [MeSH], (“hydrotherapy” [MeSH], “aquatic therapy” [MeSH], “Water-based exercise”, “Aquatic exercise”), (“elderly” [MeSH] OR “Older adults”), “effects”. For example, the following combination of terms with Boolean operators (AND/OR) was applied in Scopus: “hydrotherapy” OR “aquatic therapy” OR “water-based exercise” AND “Osteoarthritis” AND “elderly” (Supplementary Material). Additionally, relevant references within included publications and existing systematic reviews were manually searched.

### 2.2. Eligibility Criteria and Study Selection

The inclusion criteria were as follows: (1) randomized controlled trial (RCT) design; (2) water-based exercise interventions in at least one of the groups; (3) patients with OA diagnosis; (4) older people (+60 years). Publications were excluded if (1) the water-based exercise intervention was combined with other therapies; (2) the article was not written in English, Portuguese or Spanish language.

### 2.3. Study Selection

As part of the process, two authors (B.M. and S.V.) independently assessed the title and abstracts of the identified studies for eligibility, independently reviewed the full text of the potentially eligible studies, selected the works that met the inclusion criteria, and compared the results in order to reach an agreement. A third author (C.A.) was consulted if it was unclear whether a study met the selection criteria.

### 2.4. Data Extraction

Information was collected on the characteristics of the participants, exercise training programs, main outcomes, adverse events, and dropouts. In this procedure, information was taken from the original articles by one author and then collated by a second author. In cases where there was disagreement, a third author was consulted, and consensus was reached through discussion.

### 2.5. Quality Appraisal

The methodological quality of each RCT was retrieved from the Physiotherapy Evidence Database (PEDro). If a trial was not included in PEDro, two authors independently assessed its quality. Any disagreements between the authors were resolved through discussion and consensus. The quality of the studies was categorized according to the following cut-off points: excellent (9–10), good (6–8), fair (4–5), and poor (<3) [21].

## 3. Results

### 3.1. Selection Process of Studies

**Figure 1:**
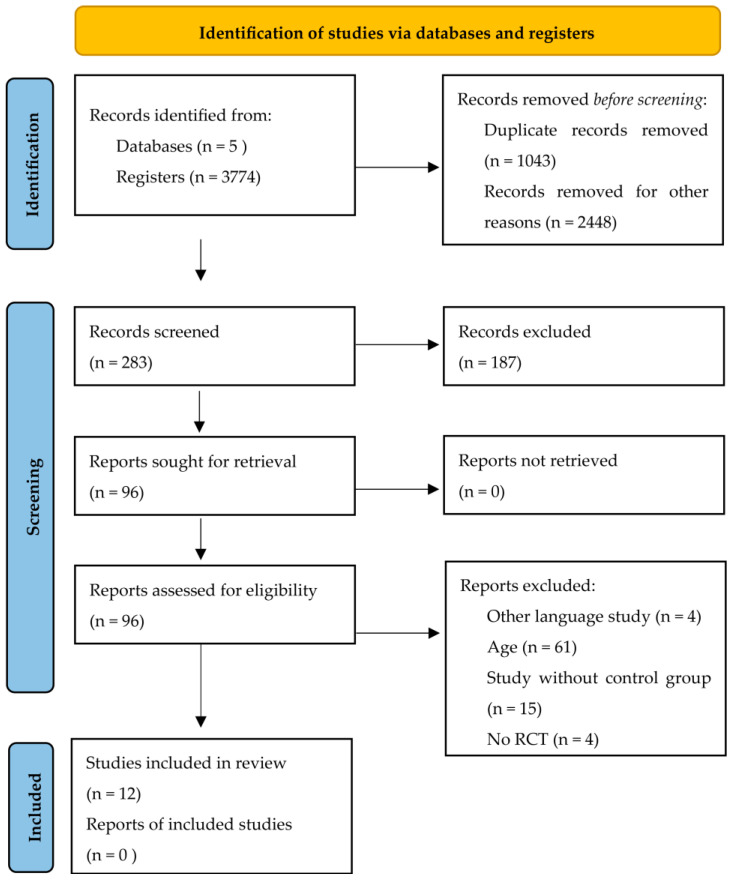
PRISMA 2020 flow diagram.

Out of an initial pool of 3774 records, 1043 duplicates were removed, and 2448 reports were excluded for not meeting the inclusion criteria. This left 283 studies selected for full-text assessment. There were 96 studies evaluated for eligibility, of which 61 did not meet the age criterion, 15 did not have a control group, 4 did not meet the randomized controlled trial criteria, and 4 did not meet the language criteria. Finally, 12 reports met the inclusion criteria and were included in the systematic review. Two sets of articles [22,23,24,25] shared the same sample and methodological design, resulting in a total of 10 investigations included in the review (Figure 1).

### 3.2. Design and Samples

The total sample size from all studies was 865 participants (excluding participants duplicated in the mentioned investigations). Sample sizes ranged between 35 [26] and 312 [27] participants. The characteristics of the participants varied among studies, with age range spanning from 60 to 83 years and the majority of the participants were female (81.5%). Six of the selected studies [24,25,28,29,30,31] included participants with knee OA, two focused on hip OA [22,23], two reported OA in both joints [27,32], and only one included patients with OA in the hip, knee, spine, and/or hands [26].

The set of studies conducted by Munukka, Waller, et al. [24,25] included participants who followed their usual medication regimen for OA. Fisken et al. [26] reported participants using analgesics (n = 17) and non-steroidal anti-inflammatory drugs (n = 7). In the study by Yennan et al. [31], participants consumed Paracetamol (500 mg) during the 6-week intervention. Hale et al. [32] noted that most participants used 2 to 4 medications (46%), 5 to 7 medications (29%), or 8 to 10 medications (15%), with only 4 participants (10%) not taking any prescribed medication. In the studies by Arnold et al. [22,23], participants used between 0 and 6 medications. The remaining publications did not provide information in this regard. All the studies were published between 2005 and 2022. Table 1 shows a summary of their main characteristics.

### 3.3. Interventions Characteristics

Five of the studies included a non-exercise control group [24,25,27,29,32,33]. Two studies compared an aquatic exercise group with a land-based exercise group [28,31]. Another study divided participants into three groups, as follows: a non-exercise control group, an aquatic exercise group, and a land-based exercise group [30]. One study split participants into an aquatic exercise group and a control group that performed seated exercise in warm water [26]. Arnold et al. [22,23] divided participants into the following three groups: an aquatic exercise group, an aquatic exercise and education group, and a control group (Table 2).

Exercise interventions lasted between 6 [31,33] and 52 [27] weeks. The studies reported frequencies ranging from two [22,23,26,27,29,32,33] to four [31] sessions per week. The duration of sessions varied from 20 to 65 mins. Munukka, Waller, Dias et al. [24,25,33] used maximal heart rate and RPE for controlled intensity. The remaining studies did not specify how exercise intensity was controlled.

### 3.4. Main Outcomes

#### 3.4.1. OA Symptoms

Eleven of the twelve studies included in this review analyzed the effects of aquatic exercise on OA specific symptoms [22,23,24,25,26,27,29,30,31,32,33]. Five of them reported significant inter-group differences. Taglietti et al. [29], Cochrane et al. [27], and Dias et al. [33] found better health scores, as assessed by the Western Ontario and McMaster Universities Arthritis Index (WOMAC), in the aquatic exercise program compared to the education group program and the non-exercise control group. However, Munukka et al. [24], who evaluated OA symptoms using the same questionnaire, found improvements only in the stiffness subscale for the aquatic exercise group. Another study found that the group performing aquatic exercise reported less pain, as assessed by the Visual Analogue Scale (VAS), compared to the land-based exercise group after a 6-week intervention [31]. Wang et al. [30] showed improvements as assessed by the KOOS in the subscales of pain, symptoms, sport/recreation, and QOL in aquatic and land-based exercise group compared to non-exercise control group.

Four of the twelve studies reported a significant intra-group improvement in OA symptoms after aquatic training intervention [27,29,30,31].

#### 3.4.2. Walking Ability

Nine studies analyzed the effects of aquatic exercise on walking ability [22,23,25,26,27,28,29,30,32]. Only two of the nine studies reported significant inter-group differences. Cochrane et al. [27] reported significant inter-group improvements after comparing a 48-session aquatic exercise program with a control group that did not participate in any exercise. In the study by Waller et al. [25], they demonstrated that walking speed was higher in aquatic training group compared to the control group after 16 weeks of intervention.

Two studies reported a significant intra-group improvement in walking ability. Wang et al. [30] and Etesami et al. [28] found improvements in both the aquatic training group and the land-based training group.

#### 3.4.3. Muscle Strength

Eight studies analyzed the effects of aquatic exercise on strength outcomes [22,23,26,27,28,31,32,33]. Cochrane et al. [27] and Dias et al. [33] reported significant inter-group improvements when comparing an aquatic exercise program with a control group that did not exercise. However, none of the remaining studies found significant inter-group differences in this variable.

Additionally, only four of the eight studies demonstrated a significant positive effect of aquatic exercise on muscle strength [27,28,31,32].

#### 3.4.4. Fear of Falling

Four studies evaluated the effects of aquatic exercise on fear of falling [22,23,26,32]. Arnold et al. [22,23] found that the aquatic exercise and education group showed a greater reduction in fear of falling scores compared to the aquatic exercise group or the control group. Additionally, the aquatic exercise and education group demonstrated a significant decrease in fear of falling compared to the baseline. However, none of the remaining studies reported significant inter-group or intra-group differences in this variable.

#### 3.4.5. Balance

Three studies evaluated balance outcomes [22,23,31]. Yennan et al. [31] showed better balance outcomes in the aquatic group compared to the land-based group. However, none of the remaining studies reported significant inter-group differences in this variable.

Two investigations showed a significant positive effect derived from this practice compared to baseline levels [22,31]. However, Arnold and Faulkner [23] did not observe any improvements in this outcome.

#### 3.4.6. Quality of Life

Quality of life was assessed using the SF-36 questionnaire (Medical Outcome Study Short Form 36-item Health Survey) in the interventions by Taglietti et al., Munukka et al., and Cochrane et al. [24,27,29]. Additionally, Cochrane et al. [27] utilized the EQ-5D questionnaire. However, none of the three studies reported significant inter-group differences in this variable.

In the intra-group analysis, only the study by Taglietti et al. [29] reported a significant improvement in the physical functioning subscale after an 8-week aquatic intervention, with sustained benefits observed at the 3-month follow-up.

#### 3.4.7. Range of Motion

Two studies evaluated the effects of aquatic exercise on range of motion [30,31]. Neither of the two studies reported significant inter-group differences.

Only Yennan et al. [31] observed an improvement in the results of the Sit-and-Reach test after a 6-week intervention in both the aquatic- and land-based exercise groups.

#### 3.4.8. Dual-Task Function

Only Arnold et al. [22,23] analyzed dual-task function using the TUG-cog test but did not report significant inter-group or intra-group differences in this variable among the aquatic exercise group, the aquatic exercise and education group, and the control group.

#### 3.4.9. Fall Risk

One study analyzed the effect of aquatic exercise on fall risk using the Physiological Profile Assessment Test [32] but did not report significant inter-group differences. However, the results showed significant improvements in contrast sensitivity and reaction time after a 12-week aquatic exercise program.

#### 3.4.10. Depressive Symptoms

Aquatic exercise did not have any significant impact on depressive symptoms according to the results reported in the only study investigating this outcome [29].

#### 3.4.11. Body Composition

One investigation explored the impact of aquatic exercise on body composition [25]. The results indicated significant improvements in the patients’ body composition after a 16-week aquatic exercise routine compared to the control group, who continued their usual leisure activities.

### 3.5. Dropouts and Adverse Events

A total of 147 dropouts were observed across the eight studies that provided information on this matter [22,24,26,27,29,30,32,33], with 75 of them occurring in the aquatic exercise groups. The primary reasons for dropouts included discontinuing the intervention and/or medical reasons. Adverse effects were observed in the experimental group in five of the included studies. Participants reported two medical consultations due to aquatic training, one case of exacerbated hip pain, two cases of increased leg pain, one instance of dizziness during exercise, and a fall resulting in spinal pain (without fracture) caused by slipping on a wet surface while entering the pool.

### 3.6. Methodological Quality

The results of the methodological quality assessment for ten RCTs were retrieved directly from the PEDro database [22,23,26,27,29,30,31,32,33], while additional evaluations were conducted for the remaining studies. The methodological quality was rated as fair in two studies [26,31] and good in the remaining studies. A full description of the quality analysis was also provided (see Table 2).

## 4. Discussion

In this review, the aim was to assess the effectiveness of water-based exercise as a rehabilitation strategy for managing OA in older adults. To ensure the highest standard of evidence, only RCTs were considered. The majority of these studies demonstrated strong methodological quality, with a few showing fair quality, which bolsters the credibility of our conclusions. The findings, along with the specific program features discussed, provide valuable insights for health professionals in developing and implementing aquatic exercise programs for elderly individuals with OA. These results also support the argument in favor of non-invasive therapies for managing OA, as previously recommended [34].

Almost all the reviewed studies examined the impact of aquatic exercise on OA symptoms, which is crucial given that their severity frequently results in disability and impaired function. This is particularly significant as no definitive treatment currently exists for OA [35]. Aquatic exercise has previously been shown to be effective in improving OA clinical symptoms such as pain, stiffness, and physical function [36,37]. However, mixed results were found in older adults with OA, with only three out of ten studies showing intra-group improvements. This aligns with the previous research highlighting the limited scientific support for the efficacy of exercise in managing pain and stiffness in older adults with OA [38,39]. In this context, aerobic exercise, strength training, or neuromuscular exercise might be more favorable alternatives [40].

Symptomatic hip/knee OA has been regarded as the strongest contributor to walking difficulty. This relationship increases with the number of joints affected [41]. In this sense, people with OA are often encouraged to walk daily to maintain functional autonomy [42]. However, according to the findings of this review, the effectiveness of prescribing aquatic exercise to improve walking ability in old people with OA remains uncertain due to mixed results. Of the nine reviewed studies, only two showed significant inter-group improvements favoring water-based exercise participants, while one study indicated positive intra-group benefits. A meta-analysis by Tanaka [43] on the efficacy of exercise therapy for enhancing walking ability in people with OA provided very-low-quality evidence that exercise increased total walking distance, and low- to moderate-quality evidence for improvements in walking time and gait velocity. These results suggest that the impact of aquatic exercise on walking ability may follow a similar trend, with walking as a form of exercise still likely being the most effective option for improving this outcome [44].

An inverse relationship between muscular strength and knee OA has been well-documented, leading to the widespread inclusion of strengthening interventions in clinical guidelines to manage knee symptoms and functional disability [45]. Muscle weakness is prevalent in individuals with hip OA [46], yet research on the effectiveness of exercise interventions for hip OA remains comparatively limited. Similarly to the findings on walking ability, the impact of water-based exercise on muscular strength in old individuals with OA yielded inconsistent results. These findings align with those of a systematic review by Mattos et al. [47], which revealed that only two of the five studies reviewed reported positive effects of aquatic exercise on muscular strength in people with OA.

This lack of consistent effectiveness may stem from two main factors. Firstly, age has been identified as a variable that negatively influences the potential benefits of water-based exercise on muscular strength. For instance, Prado et al. [48], after conducting multiple meta-analyses including both young and elderly participants, reported significant muscular strength improvements in younger groups, while the impact of aquatic exercise among older adults was limited. Secondly, exercise intensity has also been recognized as crucial for achieving strength gains. In a review of 15 studies on aquatic exercise’s effects on lower limb strength in individuals with musculoskeletal conditions, Heywood et al. [49] concluded that insufficient resistance application in water significantly contributed to the limited gains in hip and knee muscle strength. Although this idea cannot be thoroughly explored, as most of the reviewed studies did not provide detailed information on exercise intensity, it is plausible to assume that due to the participants’ age and health conditions, the exercise likely elicited only low levels of effort.

Individuals with OA, particularly those with symptomatic hip or knee OA, face an elevated risk of falls, even when accounting for common risk factors [50]. The findings of this review do not provide clear evidence that aquatic exercise has a positive effect on either fear of falling or fall risk. These results align with previous studies, suggesting limited evidence supporting the use of aquatic exercise for improving physiological components associated with fall risk [15]. The lack of clear effectiveness may be attributed to muscle weakness and impaired balance, which are key contributors to fall risk [51]. Notably, aquatic exercise did not show consistent improvements in muscular strength. However, two out of the three studies that assessed balance as an outcome did report positive changes in participants who engaged in water-based exercise. These findings might indicate that both fitness dimensions should be equally improved to reduce fall risk. In any event, it seems that water-based exercise may not be the most effective therapeutic approach for managing fall risk among elderly people with OA, as other authors have previously noted [52].

A substantial body of research has indicated that aquatic exercise interventions can significantly enhance the quality of life for individuals with and without chronic diseases [12]. However, this benefit appears less evident in older adults with OA, with only one out of three studies reporting positive intragroup changes. Several factors contribute to a reduced quality of life in older adults with OA, such as fall risk, sarcopenia, sexual health issues, and incontinence [53]. It is plausible that the reviewed aquatic interventions were not tailored to address these specific issues, thereby limiting their effectiveness.

Several key outcomes for individuals with OA, such as range of motion, depression, and body composition, were only assessed by one or two studies, with positive results primarily observed for the latter. However, the limited number of investigations restricts further analysis. In this respect, none of the reviewed studies included systemic biomarkers for tracking intervention outcomes, as previously suggested for this population [54]. On the positive side, since this review focused exclusively on RCTs, it allowed for a comparison between aquatic exercise and other therapies. The findings suggest that participating in water-based exercise programs is more effective than usual care. However, the benefits are not superior to those observed from land-based interventions, a result previously noted in people with OA [55].

The main strength of this review lies in its novelty, since it focuses exclusively on older adults with OA, and the fact that only RCTs were included for analysis. The findings suggest that aquatic exercise is a feasible therapy that can be recommended to improve balance and reduce stiffness, particularly for older adults who are inactive. In this context, performing two sessions per week, each lasting at least 20 min, and using the RPE scale to monitor exercise intensity is advised. Also, combining flexibility and strengthening exercises while using assistive devices to support balance and improve workout effectiveness is recommended to maximize the therapeutic benefits of this approach. Nevertheless, there are several limitations that need to be addressed. First, due to the heterogeneity of the reviewed data, a meta-analysis could not be conducted. This fact compromises statistical power, increases the risk of bias, and limits the generalizability of the results. Second, the reporting of exercise intensity was insufficient, which undermines the replicability of the interventions. This lack of information regarding exercise intensity is noteworthy, as it is a critical factor in providing effective exercise guidelines for the elderly population [56]. Third, the limited number of studies involving male participants precludes any analysis of potential sex differences in the outcomes. This is a notable observation, as sex is a factor that can influence the effects of exercise [57]. In addition, the variability in interventions used by comparison groups prevents further analysis on whether alternative exercise programs might be more effective than water-based therapy, leaving this topic open for further research in patients with OA. Finally, it is essential to account for the inherent limitations of the review design, such as language restrictions, exclusion of grey literature, and the risk of publication bias.

Future research recommendations include conducting larger-scale, high-quality trials to assess long-term outcomes and adherence strategies. Additionally, studies should ensure a representative male sample and focus on comparing the efficacy of aquatic exercise with land-based modalities for addressing OA symptoms, functional limitations, and quality of life, while providing comprehensive training load details (e.g., intensity, frequency, duration) and incorporating follow-up phases.

## 5. Conclusions

The effectiveness of water-based exercise programs for managing OA symptoms, improving muscular strength, balance, fear of falling, and quality of life in older adults with OA appears to be limited. Preliminary scientific evidence suggests that while aquatic exercise may be particularly effective in improving balance and managing stiffness in older adults with knee OA, it does not produce significantly greater benefits than other exercise programs in enhancing overall health in this population. However, the feasibility and high adherence associated with this form of therapy make it a viable exercise recommendation for older adults with knee OA. Future comparative studies with balanced representation of both sexes should investigate the impact of exercise intensity on the short- and long-term efficacy of aquatic therapy.

## Figures and Tables

**Table 1:** Descriptive characteristics of the included studies.

Author, Design and Country	Sample	Intervention	Outcomes	Results	Dropouts and Adverse Events
[28] Design: RCT Country: Iran	Participants (n): 54 women with knee OA Gender: 54F Age, years (range): 60–69 BMI, kg/m^2^: NR Medication: NR	Duration: 8 weeks Volume: 60 min Frequency: 3 days/week Intensity: NR EG1 Type: Aquatic training Activities: Protocol proposed by National Academy of Sports Medicine which includes 4 stages of inhibition, lengthening, activation, and integration. EG2 Type: Land Exercise Therapy Activities: Protocol proposed by National Academy of Sports Medicine which includes 4 stages of inhibition, lengthening	Walking ability ▪ 6MWT (m) ▪ TUG (s) ▪ 40- m fast-paced walk test (s) Muscle Strength ▪ 30 s sit-to-stand (rep) ▪ Stair climb test (rep)	Intra-group (*p* < 0.05) ↑ Walking ability (6MWT, TUG and 40 m fast-paced walk test) in both groups ↑ Muscle strength (30 s sit-to-stand and stair climb test) in both groups. Inter-group (*p* < 0.05)—NO	Dropouts: NR Adverse events: NR
[24] Design: RCT Country: Finland	Participants (n): 87 postmenopausal women with mild knee OA Final sample: n = 85 (EG: 42; CON: 43) After follow-up: 77 (EG: 40; CON: 37) Gender: EG: 43F; CON: 44F Age, years (mean; SD; range): EG: 64 ± 2; CON: 64 ± 2; 60–68 BMI, kg/m^2^ (mean; SD): EG: 26.6 ± 3.8; CON: 27.1 ± 3.5 Medication, yes/no: EG: 11/43; CON: 9/44	Duration: 16 weeks (+12-month follow-up) EG Type: Aquatic resistance training Activities: Supervised lower limb aquatic resistance training in small groups (6–8 participants). Three resistance levels were used to ensure intensity, and the movements were performed without contact with the pool walls or floor. Volume: 60 min Frequency: 3 days/week Intensity: RPE: 13–17 (Borg 6–20); HR_max_: 85 ± 8–9%; Average HR: 61 ± 6.3% CON Maintained usual care and were asked to continue their habitual leisure time activities. They were offered the possibility of participating in 2 sessions consisting of 1 h of light stretching and relaxation during the intervention period.	Quality of life: ▪ SF-36 (score) ➢ Physical Functioning ➢ Role-Physical ➢ Bodily Pain ➢ General Health ➢ Vitality ➢ Social Functioning ➢ Role-Emotional ➢ Mental Health OA symptoms: ▪ WOMAC (score) ➢ Pain ➢ Stiffness ➢ Physical Function	Intra-group (*p* < 0.05)—NR Inter-group (*p* < 0.05) < WOMAC stiffness score in EG compared to CON (−8.5 mm (−14.9 to −2.0)) after intervention.	Dropouts Intervention period: EG: Withdrew (n = 1) CON: Withdrew (n = 1) Follow-up period: EG: Died (n = 1) Hip arthroplasty (n = 1) CON: Died (n = 1) Cancer (n = 1) Ménière’s disease (n = 1), Withdrew (n = 2) Adverse events: 2 medical consultations (bilateral knee pain and dyspnea) as a result of the aquatic training
[29] Design: RCT Country: Brazil	Participants (n): 60 participants with knee OA Final sample: n = 49 (EG1: 28; EG2: 21) Gender: EG1: 23F + 8M; EG2: 18F + 11M Age, years (mean; SD): EG1: 67.3 ± 5.9; EG2: 68.7 ± 6.7 BMI, kg/m^2^ (mean; SD): EG1: 29.2 ± 0.8; EG2: 30.4 ± 0.9 Medication: NR	Duration: 8 weeks (+3-month follow-up) Intensity: NR EG1 Type: Aquatic Program Activities: 5 min warm-up, followed by leg stretches. It included 15 min of knee and hip exercises with elastic bands, 20 min of aerobic activity, 10 min of step and balance exercises, and ended with a 10 min cool down. Volume: 60 min Frequency: 2 days/week EG2 Type: Education group program Activities: Maximum of 5 participants per group. The strategies focused on pain control, physical exercise, nutrition, weight management, medication, balance, proprioception, fall prevention, and chronic pain management. Additionally, received home exercise guidelines, to be practiced 2–3 days/week. Included warm-up routines, self-stretching, isometric, and dynamic movements, as well as proprioceptive and functional exercises for the lower limbs, and cool-down. Volume: 120 min Frequency: 1 day/week	Quality of life: ▪ SF-36 (score) ➢ Physical Functioning ➢ Role-Physical ➢ Bodily Pain ➢ General Health ➢ Vitality ➢ Social Functioning ➢ Role-Emotional ➢ Mental Health OA symptoms: ▪ WOMAC (score) ➢ Pain ➢ Stiffness ➢ Physical Function ▪ Pain–VAS (cm) Walking ability ▪ TUG (s) Depressive symptoms ▪ Yesavage Geriatric Depression Scale (score)	Intra-group (*p* < 0.05) ↑ SF-36 physical functioning score in EG1 after intervention and follow-up (MD (95% CI): −9.6 (2.9 to 16.3) and 10.6 (3.6 to 17.8)) ↓ WOMAC total score in EG1 after intervention and follow-up (MD (95% CI): −11 (−14.9 to −9.6) and −11.8 (−19.3 to −3.6)) ↓ WOMAC pain score in EG1 after intervention and follow-up (MD (95% CI): −3.3 (−6.5 to −0.1) and −3.1 (−6.3 to −0.03)) Inter-group (*p* < 0.05) < WOMAC total score in EG1 compared to EG2 after intervention and follow-up (MD (95% CI): −14.2 (−18 to −10.5) and −12.3 (−24.6 to −6.1)) < WOMAC pain score in EG1 compared to EG2 after intervention (MD (95% CI): −3.8 (−8.7 to −1))	Dropouts EG1: Health problems (n = 2); Dropped out of the sessions (n = 1) EG2: Discontinued intervention (n = 8) Adverse events: NO
[25] **Design:** RCT **Country:** Finland	Participants (n): 87 postmenopausal women with mild knee OA Final sample: n = 84 (EG: 42; CON: 42) After follow-up: 76 (EG: 40; CON: 36) Gender: EG: 43F; CON: 44F Age, years (mean; SD; range): EG: 63.8 ± 2.4; CON: 63.9 ± 2.4; 60–68 BMI, kg/m^2^ (mean; SD): EG: 26.6 ± 3.8; CON: 27.1 ± 3.5 Medication, yes/no: EG: 11/43; CON: 9/44	Duration: 16 weeks (+12-month follow-up) EG Type: Aquatic resistance training Activities: Supervised lower limb aquatic resistance training in small groups (6–8 participants). Three resistance levels were used to ensure intensity, and the movements were performed without contact with the pool walls or floor. Volume: 60 min Frequency: 3 days/week Intensity: RPE: 13–17 (Borg 6–20); HR_max_: 85 ± 8–9%; Average HR: 61 ± 6.3% CON Maintained usual care and were asked to continue their habitual leisure time activities. They were offered the possibility of participating in 2 sessions consisting of 1 h of light stretching and relaxation during the intervention period.	OA symptoms: ▪ KOSS (score) ➢ Pain ➢ Symptoms ➢ Activities of daily living ➢ Sports and recreation ➢ Quality of life Walking ability ▪ UKK 2 km walking test (m/s) Body composition ▪ Fat mass (kg) ▪ Body mass (kg) ▪ Lean mass (kg) ▪ BMI (kg/m^2^)	Intra-group (*p* < 0.05)—NR Inter-group (*p* < 0.05) > Walking speed in EG compared to CON (1.83 ± 0.16 vs. 1.73 ± 0.17 m/s) after intervention and after follow-up (1.82 ± 0.14 vs. 1.77 ± 0.13 m/s). < Fat mass in EG compared to CON (24.8 ± 8.8 vs. 26.4 ± 8.1 kg) after intervention < Body mass in EG compared to CON (68.2 ± 10.4 vs. 70.9 ± 11.3 kg) after intervention < BMI in EG compared to CON (26.2 ± 3.9 vs. 27.1 ± 3.6 kg/m^2^) after intervention	Dropouts Intervention period: EG: Withdrew (n = 1) CON: Withdrew (n = 1) Did not attend DXA (n = 1) Follow-up period: EG: Died (n = 1) Hip arthroplasty (n = 1) CON: Died (n = 1) Cancer (n = 1) Ménière’s disease (n = 1), Withdrew (n = 2) Adverse events: 2 medical consultations (bilateral knee pain and dyspnea) as a result of the aquatic training
[33] Design: RCT Country: Brazil	Participants (n): 73 women with knee OA Final sample: n = 65 (EG: 33; CON: 32) Age, years (mean; SD): EG: 70.8 ± 5.0 CON: 71.0 ± 5.2 BMI, kg/m^2^ (mean; SD): EG: 30.5 ± 4.3 CON: 30.0 ± 5.2 Medication: NR	Duration: 6 weeks EG Type: Hydrotherapy + education program Activities: Supervised lower limb strengthening exercises that included closed kinetic chain exercises using floats as well as multidirectional walking tasks. Educational information (lecture) about knee OA during daily activities (diagnosis, symptoms, prognosis and basic care) given by an investigator. Volume: 40 min Frequency: 2 days/week Intensity: moderate (RPE; Borg scale) CON Educational information (lecture) about knee OA during daily activities (diagnosis, symptoms, prognosis, and basic care) given by an investigator. They also received weekly advice via telephone during six consecutive weeks.	OA symptoms: ▪ WOMAC (score) ➢ Pain ➢ Stiffness ➢ Physical Function Muscle Strength ▪ Muscle Strength (flexion/extension) ▪ Muscle Power (flexion/extension) ▪ Muscle Resistance (flexion/extension)	Intra-group (*p* < 0.05)—NR Inter-group (*p* < 0.05) < WOMAC pain score in EG compared to CON after intervention (MD (95% CI): 10.9 (18 to 3) < WOMAC physical function score in EG compared to CON after intervention (MD (95% CI): 11.9 (18 to 5) > Muscle Strength (flexion and extension) in EG compared to CON after intervention [Flexion: (MD (95% CI): 4.9 (0.2 to 9); Extension: (MD (95% CI): 7.3 (0.0006 to 14)] > Muscle Power (flexion) in EG compared to CON after intervention (MD (95% CI): 5.0 (0.3 to 9) > Muscle Resistance (extension) in EG compared to CON after intervention (MD (95% CI): 4.8 (0.3 to 9)	Dropouts Intervention period: EG: Food poisoning (n = 1) Surgery (n = 1) Being uncontactable (n = 2) CON: Transport (n = 1) Health problems (n = 2) Being uncontactable (n = 1) Adverse events: NO
[26] Design: RCT Country: New Zealand	Participants (n): 35 participants with OA in the hips, knees, spine and/or hands Final sample: n = 25 (EG1: 13; EG2: 12) Gender: EG1: 18F + 1M; EG2: 15F + 1M Age, years (mean; SD): EG1: 71.9 ± 7.3; EG2: 70.4 ± 6.5 BMI, kg/m^2^: NR Medication, yes/no: Analgesics: EG1: 9/13; EG2: 8/12 Non-steroidal anti-inflammatory drugs: EG1: 3/13; EG2: 4/12	Duration: 12 weeks Intensity: NR EG1 Type: Aquatic program Activities: Aquatic aerobic and strength-training exercises. Supervised in small groups (max 10 participants) used music to motivate, synchronize, and pace movement velocity. Beats per minute helped quantify velocity, and the instructor encouraged greater movement range as the program progressed. Volume: 45–60 min Frequency: 2 days/week EG2 Type: Arthritis Foundation Arthritis Water Exercise DVD Activities: Seated exercises in warm water were chosen to help alleviate acute OA symptoms, serving as a motivator for attendance. The focus was on range of motion and relaxation, with an experienced aqua instructor overseeing sessions, limited to 10 participants. Volume: 35–40 min Frequency: 1 day/week	OA symptoms: ▪ AIMS2-SF (score) ➢ Physical ➢ Symptoms ➢ Affects Walking ability ▪ TUG (s) ▪ 400 m walk test (s) Muscle Strength ▪ Handgrip dynamometry (kg) ▪ 15 s step test (rep) ▪ 30 s sit-to-stand (rep) Fear of falling ▪ FES-I (score)	Intra-group (*p* < 0.05)—NO * Inter-group (*p* < 0.05)—NO *	Dropouts EG1: Unrelated illness (n = 4); Experienced hip pain (n = 1); Found pool too cold (n = 1) EG2: Unrelated illness (n = 2); Lack of time (n = 1); Surgery brought forward (n = 1) Adverse events: 1 participant reported exacerbation of hip pain
				* Based on intention–to-treat analysis	
[32] Design: RCT Country: New Zealand	Participants (n): 39 participants with OA in the hip and/or knee Final sample: n = 35 (EG: 20; CON: 15) Gender: EG: 17F + 6M; CON: 12F + 4M Age, years (mean; SD; range): EG: 75.7 ± 1.1; CON: 73.6 ± 1.5; 70.5–77.9 BMI, kg/m^2^: NR Medication: Most participants used 2 to 4 (46%), 5 to 7 (29%), or 8 to 10 medications (15%). Only 4 participants (10%) took no prescribed medication.	Duration: 12 weeks Intensity: NR EG Type: Water-based exercise Activities: Groups of 10–12 participants. Exercise sessions included warm-up, warm-down, balance exercises and cold down. Exercises involved starting with eyes open, then closed, using a pool noodle for balance, later removing it. Noodles, dumbbells, flutter boards, and partner exercises were used to challenge balance with added resistance and disturbances. Volume: 20–60 min Frequency: 2 days/week CON Type: Computer-skills training program Activities: Received individualized training starting at their own skill level seated in front of a computer from instructors of a similar age. Instructors would circulate among participants, providing feedback while participants practiced their skills Volume: 60 min Frequency: 2 days/week	OA symptoms: ▪ WOMAC (score) ➢ Pain ➢ Stiffness ➢ Physical Function ▪ AIMS2-SF (score) ➢ Physical ➢ Symptoms ➢ Affects Walking ability ▪ TUG (s) Muscle Strength ▪ 15 s step test (rep) Fear of falling ▪ ABC Scale (score) Fall risk ▪ PPA (score) ➢ Contrast sensitivity ➢ Reaction time (s) ➢ Proprioception (deg) ➢ Strength (kg) ➢ Sway on foam, anterior-posterior (mm) ➢ Sway on foam, medial-lateral (mm)	Intra-group (*p* < 0.05) ↑ Step Test results in EG (Change = left leg: 2.1 ± 2.3; right leg step 1.7 ± 2.4) ↑ Step Test results in CON (Change = left leg: 2.1 ± 2; right leg step 1.4 ± 2) ↑ Contrast sensitivity in CON (−1.43, −2.35 to −0.50) ↑ Reaction time in CON (86.83, 9.86 to 163.79) Inter-group (*p* < 0.05)—NO	Dropouts EG: Stroke (n = 1); Total knee/hip replacement (n = 2) CON: Husband fell ill so no longer had transport (n = 1) Adverse events: 2 participants reported exacerbation of leg pain
[30] Design: RCT Country: Taiwan	Participants (n): 84 participants with knee OA Final sample: n = 78 (EG1: 26; EG2: 26; CON: 26) Gender: EG1: 22F + 4M; EG2: 23F + 3M; CON: 22F + 4M Age, years (mean; SD): EG1: 66.7 ± 5.6; EG2: 68.3 ± 6.4; CON: 67.9 ± 5.9 BMI, kg/m^2^ (mean; SD): EG1: 26.6 ± 2.5; EG2: 25.4 ± 2.4; CON: 26.6 ± 2.08 Medication: NR	Duration: 12 weeks Volume: 60 min Frequency: 3 days/week Intensity: NR EG1 Type: Aquatic exercise program Activities: The main components included flexibility and aerobic training. Based on the Arthritis Foundation Aquatics Program, focused on joints in the trunk, shoulders, arms, and legs, targeting upper and lower limb muscles, balance, and coordination. Used speed, surface area, movement direction, and water turbulence to adjust resistance and intensity. EG2 Type: Land-based exercise program Activities: The main components include flexibility and aerobic training. Based on the People with Arthritis Can Exercise Program, focused on joints in the trunk, shoulders, arms, and legs, targeting upper and lower limb muscles, balance, and coordination. Used movement against gravity and variations in speed, level of leg or arm raising, or moving both to create different levels of training intensity. CON Without exercise	OA symptoms: ▪ KOSS (score) ➢ Pain ➢ Symptoms ➢ Activities of daily living ➢ Sports and recreation ➢ Quality of life Walking ability ▪ 6MWT (m) Range of motion ▪ Knee flexion and extension-Goniometer (°)	Intra-group (*p* < 0.05)— ↑ Improvement in pain (KOOS) in both EG groups ↑ Walking ability (6MWT) in both EG groups Inter-group (*p* < 0.05)— > Improvement in KOOS (Pain, symptoms, sport/recreation and QOL) in both groups compared to CON	Dropouts EG1: Due to having a herpes flare-up (n = 1); Due to travel (n = 1) EG2: Due to not being interested in land exercise (n = 1); Other obligations (n = 1) CON: Admitted to a hospital for treating pneumonia (n = 2) Adverse events: 2 participants in EG2 reported increased pain after exercise, and 1 EG1 participant reported feeling dizziness during exercise
[22] Design: RCT Country: Canada	Participants (n): 54 participants with hip OA Final sample: n = 42 (EG1: 23; EG2: 19) Gender: EG1: 20F + 8M; EG2: 20F + 6M Age, years (mean; SD): EG1: 73.2 ± 4.8; EG2:74.4 ± 7.5 BMI, kg/m^2^ (mean; SD): EG1: 29.2 ± 5.2; EG2:30.4 ± 4.5 Medication (number): EG1: 2.9 ± 2.6; EG2: 2.9 ± 2.5	Duration: 11 weeks Volume: 45 min Frequency: 2 days/week Intensity: NR EG1 Type: Water exercise program + education Activities: Warm-up, strengthening exercises (using floats, noodles, sponges, paddles for resistance), trunk control, posture and balance activities, and a cool-down. Additionally, participants attended a 30 min educational session before one aquatic class each week. EG2 Type: Water exercise program Activities: Warm-up, strengthening exercises (using floats, noodles, sponges, paddles for resistance), trunk control, posture and balance activities, and a cool-down.	OA symptoms: ▪ AIMS2 (score) ➢ Physical ➢ Symptoms ➢ Affects Walking ability ▪ 6MWT (m) Dual-Task Function ▪ TUG_cog_ (s) Balance ▪ Berg Balance Scale (score) ▪ MCTSIB (s) Muscle Strength ▪ 30 s sit-to-stand (rep) Fear of falling ▪ ABC Scale (score)	Intra-group (*p* < 0.05) ↑ Improvement in MCTSIB in EG1 ↑ Improvement in ABC Scale in EG1 Inter-group (*p* < 0.05)—NO	Dropouts EG1: Mobility (n = 1); Medical (n = 1); Personal (n = 1); Transportation (n = 1); Surgery (n = 1) EG2: Medical (n = 2); Pain (n = 2); Allergy (n = 1); Surgery (n = 2) Adverse events: 1 moderate adverse event occurred in EG2—a fall causing spinal pain, but no fracture—due to slipping on a wet surface while entering the pool.
[23] Design: RCT Country: Canada	Participants (n): 79 participants with hip OA Final sample: n = 63 (EG1: 23; EG2: 19; CON: 21) Gender: EG1: 20F + 8M; EG2: 20F + 6M; CON: 16F + 9M Age, years (mean; SD): EG1: 73.2 ± 4.8; EG2: 74.4 ± 7.5; CON: 75.8 ± 6.2 BMI, kg/m^2^ (mean; SD): EG1: 29.2 ± 5.2; EG2:30.4 ± 4.5; CON: 30 ± 5.7 Medication (number): EG1: 2.9 ± 2.6; EG2: 2.9 ± 2.5; CON: 3.2 ± 2.8	Duration: 11 weeks Volume: 45 min Frequency: 2 days/week Intensity: NR EG1 Type: Water exercise program + education Activities: Warm-up, strengthening exercises (using floats, noodles, sponges, paddles for resistance), trunk control, posture and balance activities, and a cool-down. Additionally, participants attended a 30 min educational session before one aquatic class each week. EG2 Type: Water exercise program Activities: Warm-up, strengthening exercises (using floats, noodles, sponges, paddles for resistance), trunk control, posture and balance activities, and a cool-down. CON Continued with their usual activities and were asked to not begin an exercise program	OA symptoms: ▪ AIMS2 (score) ➢ Physical ➢ Symptoms ➢ Affects Walking ability ▪ 6MWT (m) Dual-Task Function ▪ TUG_cog_ (s) Balance ▪ Berg Balance Scale (score) Muscle Strength ▪ 30 s sit-to-stand (rep) Fear of falling ▪ ABC Scale (score)	Intra-group (*p* < 0.05) ↑ Improvement in ABC Scale in EG1 Inter-group (*p* < 0.05) > Improvement in 30 s sit-to-stand in EG1 vs. EG2 and CON (Change: 1.5 ± 2 vs. 0.6 ± 1.7 and 0.6 ± 1.7) > Improvement in ABC Scale in EG1 vs. EG2 and CON (Change: 5.8 ± 12.4 vs. 0.8 ± 21.1 and 2.4 ± 10.7)	Dropouts EG1: Mobility (n = 1); Medical (n = 1); Personal (n = 1); Transportation (n = 1); Surgery (n = 1) EG2: Medical (n = 2); Pain (n = 2); Allergy (n = 1); Surgery (n = 2) CON: Medical (n = 2); Personal (n = 1); Deceased (n = 1); Surgery (n = 1); Transportation (n = 1) Adverse events: 1 moderate adverse event occurred in EG2—a fall causing spinal pain, but no fracture—due to slipping on a wet surface while entering the pool.
[31] Design: RCT Country: Thailand	Participants (n): 50 women with knee OA Gender: 50F Age, years (mean; SD): EG1: 65.6 ± 4.9; EG2: 66.4 ± 4.4 BMI, kg/m^2^ (mean; SD): EG1: 24.4 ± 2.7; EG2: 23.7 ± 2.2 Medication (mean rank; sum of ranks): Paracetamol (500 mg) EG1: 23.74 ± 593.5; EG2: 27.26 ± 681.5	Duration: 6 weeks Volume: 65 min Frequency: 4 days/week Intensity: NR EG1 Type: Aquatic program Activities: 10 min warm-up (stretching hamstrings, adductors, calves, and slow walking), 45 min of exercise (squats, calf raises, leg kicks, stretches, stationary cycling, fast walking), and a 10 min cool-down (slow walking). In the last three weeks, single-leg squats and calf raises were added. The warm-up and pool-down were performed beside the pool. All other exercises were performed in the pool with light water flow. EG2 Type: Land-based program Activities: 10 min warm-up (stretching hamstrings, adductors, calves, and slow walking), 45 min of exercise (squats, calf raises, leg kicks, stretches, stationary cycling, fast walking), and a 10 min cool-down (slow walking). In the last three weeks, single-leg squats and calf raises were added. Exercise on normal floor at home.	OA symptoms: ▪ WOMAC (score) ➢ Pain ➢ Stiffness ➢ Physical Function ▪ KOSS (score) ➢ Pain ➢ Symptoms ➢ Activities of daily living ➢ Sports and recreation ➢ Quality of life ▪ Pain–VAS (score) Balance ▪ COP (open/close eyes) ➢ Medio-lateral ➢ antero-posterior Muscle Strength ▪ 30 s sit-to-stand (rep) Range of motion ▪ Sit-and-reach test (cm)	Intra-group (*p* < 0.05) ↓ WOMAC, KOSS and VAS scores in both groups ↑ Range of motion in both groups ↑ 30 s sit-to-stand rep in both groups ↑ Double-leg stance COP with eyes opened or closed in antero-posterior direction in EG1 Inter-group (*p* < 0.05) < Pain (VAS) in EG1 compared to EG2 > 30 s sit-to-stand rep in EG1 compared to EG2 > Reduce double-leg stance with closed eyes in medio-lateral direction in EG1 compared to EG2. > Reduce single-leg stance in EG1 compared to EG2. > Reduce right single-leg stance with closed eyes in antero-posterior direction in EG1 compared to EG2. > Reduce left single-leg stance with opened eyes in antero-posterior direction in EG1 compared to EG2. > Reduce left single-leg stance with closed eyes in anteroposterior direction in EG1 compared to EG2.	Dropouts: NO Adverse events: NR
[27] Design: RCT Country: UK	Participants (n): 312 participants with OA in the hip and/or knee Final sample: n = 231 (EG: 111; CON: 120) Gender: 148F + 83M Age, years (mean; SD): 69.53 ± 5.98 BMI, kg/m^2^ (mean; SD): 29.67 ± 5.08 Medication (number): NR	Duration: 52 weeks, 48 sessions due to holidays (+6-months of follow-up) EG Type: Water exercise intervention Activities: Maximum of 30 participants. Warm-up, lower limb strengthening, lower limb ROM, lower limb stretches, general cardiovascular conditioning, general balance and coordination, free use of floats/individual exercises/swimming. Volume: 60 min Frequency: 2 days/week Intensity: NR CON Continued with their usual activities. Subjects were contacted quarterly by the same researcher to monitor symptoms, exercise behavior, and treatment changes.	Quality of life: ▪ SF-36 (score) ➢ Physical Functioning ➢ Role-Physical ➢ Bodily Pain ➢ General Health ➢ Vitality ➢ Social Functioning ➢ Role-Emotional ➢ Mental Health ▪ EQ-5D (score) OA symptoms: ▪ WOMAC (score) ➢ Pain ➢ Stiffness ➢ Physical Function Walking ability ▪ Eight-foot walk (m) Muscle Strength ▪ Maximal isometric quadriceps and hamstrings (kg) ▪ Ascend and descend a set of four stairs (rep)	Intra-group (*p* < 0.05) ↓ WOMAC scores in EG ↑ Ascend and descend stairs repetitions in EG Inter-group (*p* < 0.05) < WOMAC scores in EG compared to CON > Improvement in eight-foot walk in EG compared to CON > Improvement in ascend and descend stairs in EG compared to CON	Dropouts Intervention period: EG: 42 Did not give reason (n = 7) Too busy (n = 6) Disability or illness (n = 15) Death (n = 1) Lost interest (n = 7) Moved from area (n = 3) Afraid of water (n = 2) Unable to contact (n = 1) CON: 39 Did not give reason (n = 5) Too busy (n = 8) Disability or illness (n = 8) Death (n = 5) Lost interest (n = 5) Moved from area (n = 7) Unable to contact (n = 1) Follow-up period A further 18 participants withdrew Adverse events: NR

>: greater; <: lower; ↑: increment; ↓: decrement; *: Based on intention–to-treat analysis, ABC Scale: Activity-specific Balance Confidence Scale; AIMS2-SF: Arthritis Impact Measurement Scale 2-Short Form; BMI: body mass index; CON: control group; COP: postural sway; EQ-5D: EuroQol 5 Dimensions; F: female; FES-I: Falls Efficacy Scale-International; HR: heart rate; KOSS: Knee Injury and Osteoarthritis Outcome Score; M: male; MCTSIB: Modified Clinical Test of Sensory Interaction and Balance; NO: not observed; NR: not reported; OA: osteoarthritis; PA: physical activity; PASE: Physical Activity Scale for the Elderly; PPA: physiological profile assessment; QOL: quality of life; RAPA: Rapid Assessment of Physical Activity; RPE: rate of perceived exertion; TUG: timed up-and-go; VAS: Visual Analog Scale; WOMAC: Western Ontario and McMaster Universities Arthritis Index.

**Table 2:** Results of the methodological evaluation of the included studies (PEDro scale).

Author	PEDro Item										Score	Quality
	1	2	3	4	5	6	7	8	9	10		
[28]	+	−	+	−	−	−	+	+	+	+	6/10	Good
[24]	+	+	+	−	−	−	+	−	+	+	6/10	Good
[29]	+	+	+	−	−	−	+	+	+	+	7/10	Good
[25]	+	−	+	−	−	−	+	+	+	+	6/10	Good
[33]	+	+	+	−	−	+	+	−	+	+	7/10	Good
[26]	+	−	+	−	−	−	−	+	+	+	5/10	Fair
[32]	+	+	+	−	−	+	+	+	+	+	8/10	Good
[30]	+	+	+	−	−	+	+	−	+	+	7/10	Good
[22]	+	−	+	−	−	+	−	+	+	+	6/10	Good
[23]	+	+	+	−	−	+	−	−	+	+	6/10	Good
[31]	+	−	+	−	−	−	+	−	+	+	5/10	Fair
[27]	+	+	+	−	−	+	−	+	+	+	7/10	Good

1: random allocation; 2: concealed allocation; 3: baseline comparability; 4: blind subjects; 5: blind therapists; 6: blind assessors; 7: adequate follow-up; 8: intention-to-treat analysis; 9: between-group comparisons; 10: point estimates and variability.

## Supplementary Materials

The following supporting information can be downloaded at: https://www.mdpi.com/article/10.3390/geriatrics10010012/s1, Supplementary Data: Summary of search strategy.
