# Supplementary material for: Effects of Aquatic Exercise in Older People with Osteoarthritis: Systematic Review of Randomized Controlled Trials
Source: Geriatrics (Basel). 2025 Jan 13;10(1):12. doi: 10.3390/geriatrics10010012 (PMC11755622; doi:10.3390/geriatrics10010012)
Supplement: Supplementary file 1 [file geriatrics-10-00012-s001.zip › geriatrics-3296061-supplementary.pdf]

Supplementary Data

Summary of search strategy

1. Osteoarthritis (MeSH terms)

2. Older people (All fields)

3. Elderly (MeSH terms)

6. Hydrotherapy (MeSH terms)

7. Aquatic therapy (MeSH terms)

8. Water-based exercise (All fields)

9. Aquatic exercise (All fields)

10. Effects (All fields)

11. 1 AND 2 OR 3 AND 6 OR 7 OR 8

12. 1 AND 2 OR 3 AND 8 OR 9 AND 10
